# Boolean Network Model for Cancer Pathways: Predicting Carcinogenesis and Targeted Therapy Outcomes

**DOI:** 10.1371/journal.pone.0069008

**Published:** 2013-07-26

**Authors:** Herman F. Fumiã, Marcelo L. Martins

**Affiliations:** 1 Departamento de Física, Universidade Federal de Viçosa, Viçosa, Minas Gerais, Brazil; 2 National Institute of Science and Technology for Complex Systems, Rio de Janeiro, Brazil; University of California, Irvine, United States of America

## Abstract

A Boolean dynamical system integrating the main signaling pathways involved in cancer is constructed based on the currently known protein-protein interaction network. This system exhibits stationary protein activation patterns – attractors – dependent on the cell's microenvironment. These dynamical attractors were determined through simulations and their stabilities against mutations were tested. In a higher hierarchical level, it was possible to group the network attractors into distinct cell phenotypes and determine driver mutations that promote phenotypic transitions. We find that driver nodes are not necessarily central in the network topology, but at least they are direct regulators of central components towards which converge or through which crosstalk distinct cancer signaling pathways. The predicted drivers are in agreement with those pointed out by diverse census of cancer genes recently performed for several human cancers. Furthermore, our results demonstrate that cell phenotypes can evolve towards full malignancy through distinct sequences of accumulated mutations. In particular, the network model supports routes of carcinogenesis known for some tumor types. Finally, the Boolean network model is employed to evaluate the outcome of molecularly targeted cancer therapies. The major find is that monotherapies were additive in their effects and that the association of targeted drugs is necessary for cancer eradication.

## Introduction

Cancer is a genetic disease derived, with few exceptions, from mutations on single somatic cells that disregard the normal control of proliferation, invade adjacent normal tissues, and give rise to secondary tumors (metastasis) on sites different from its primary origin [1]. In the human population, cancer refers to more than 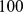 forms of a disease that can develop in almost every tissue in the body [2]. Today, cancer replaced heart disease as the leading cause of death among the United States citizens younger than 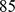 years [3] and will probably become the leading one in some other parts of the world within a few years [4]. Altough each cancer type has unique features, all these diverse tumors evolve according to a universal scheme of progression [5] which involves genetic and epigenetic events as well as an intricate network of interactions among cancer cells and their host microenvironment (stromal cells and extracellular matrix).

The tumor growth is intrinsically multiscale in nature. It involves phenomena occurring over a variety of spatial scales ranging from tissue (for instance, invasion and angiogenesis) to molecular length scales (for example, mutations and gene silencing), while the timescales vary from seconds for signaling to years for tumor doubling times. Moreover, all those processes are strongly coupled. Indeed, an oncogene activation may confer a proliferative advantage to a given cell, promoting its clonal expansion and the depletion of the nutrient and oxygen supply which, in turn, affect the growth of cell clones. To survive in a hypoxic (low level of oxygen) environment, the transformed cells may acquire new traits such as resistance to apoptosis by a tumor suppressor gene inactivation or activated synthesis of growth factors that stimulate angiogenesis. Thus information flows not only from the finer to coarser scales, but between any pair of scales [6].

Despite the extensive information on the genetic and molecular basis of cancer currently available, the integration of this information into the physiological environment of the functioning cell and tissue remains a major challenge. Due to the complexity and redundancy of tumor survival and growth pathways, increasing resistance and tumor progression still is the rule for patients with advanced cancers. Better diagnostic and effective anticancer therapies demand a fundamentally systemic understanding of the disease, starting from the molecular level. There, complexity emerges from the large number of interacting proteins and the cross-talk between diverse cell signaling pathways. This vast network of complexity, characterized by multiple feedback loops, will not be understood by merely describing all its component pathways. An integrative approach considering simplified cell-wide models of protein interactions dependent on external environmental signals and accumulated genetic alterations is demanded. In addition, modeling protein interaction networks in cancer is essential to construct the “microscopic” (molecular) level in multiscale models of tumor growth [6].

Concerning the modeling of genetic interactions, Boolean networks is a promising framework [7]. Instead of providing quantitatively precise dynamical trajectories taken by complex networks, this class of discrete systems, with binary states, qualitatively predict the sequences of states accessed by these networks along their temporal evolution. This is especially true for signaling and regulatory circuits where activation and inhibition are the basic responses. Furthermore, Boolean models use much less parameters, such as biochemical reaction rates or binding affinities, often hard to measure, than do traditional differential equations approach. Successful applications of Boolean networks in biology include the reproduction of the yeast *S. Pombe*'s cell-cycle [8], the mammalian cell cycle [9], the course of cell differentiation in early embryogenesis [10], the signaling mechanisms underlying T cell activation [11], and the behavior of the apoptotic pathway [12], [13].

In this study, we construct a Boolean network model integrating the main signaling pathways involved in cancer. These pathways and the network interconnecting them are discussed in the next subsection. Established the network topology, its dynamics is defined and validated in subsequent subsections. Then, the dynamical attractors, their stability to mutations, and the network's response to targeted “attacks” are reported in the following section. These results are interpreted in terms of the mutational events leading to carcinogenesis and cancer cell response to molecularly targeted agents. Finally, we conclude with a discussion where these biological interpretation is emphasized and confronted with experimental data on cancer genome and oncogenesis.

### The Main Cancer Pathways

In a seminal paper [14], Hanahan and Weinberg proposed a logical framework for understand the diversity and complexity of cancer. The key notion is that along the multistep process of tumor pathogenesis, normall cells need to acquire six biological traits in order to ultimately become malignant. These are the capabilities of “sustaining proliferative signaling, evading growth suppressors, resisting cell death, enabling replicative immortality, inducing angiogenesis, and activating invasion and metastasis” [15]. The acquisition of such traits is ensured by genome instability. All genes are potentially subjected to mutations. Thereby, there are several alternative ways to achieve the same cell phenotypic transformation. But there are many fewer signaling pathways controlling cell response than genes. Rather than individual genes, it seems more appropriate focusing on pathways that have a role in many tumors [16]. They include those involving receptor tyrosine kinase (RTKs), phosphatidylinosital 3-kinase (PI3K)/AKT, WNT/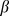-Catenin, transforming growth factor-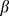 (TGF-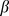)/Smads, retinoblastoma protein (Rb), hypoxia-inducible transcription factor (HIF-1), p53 and ataxia-telangiectasia mutated (ATM)/ataxia-telangiectasia and Rad3-related (ATR) protein kinases. These major pathways regulating cell death and proliferation share some genes and exhibit a substantial cross-talk among them. Some pathways were detailed studied in isolation [12], [17], [18], but in order to integrate them a natural organizing principle is represent these pathways as a network. Given the complexity and lacunas present in its structure, an operational alternative is work with a simplified model network.

**Figure 1:**
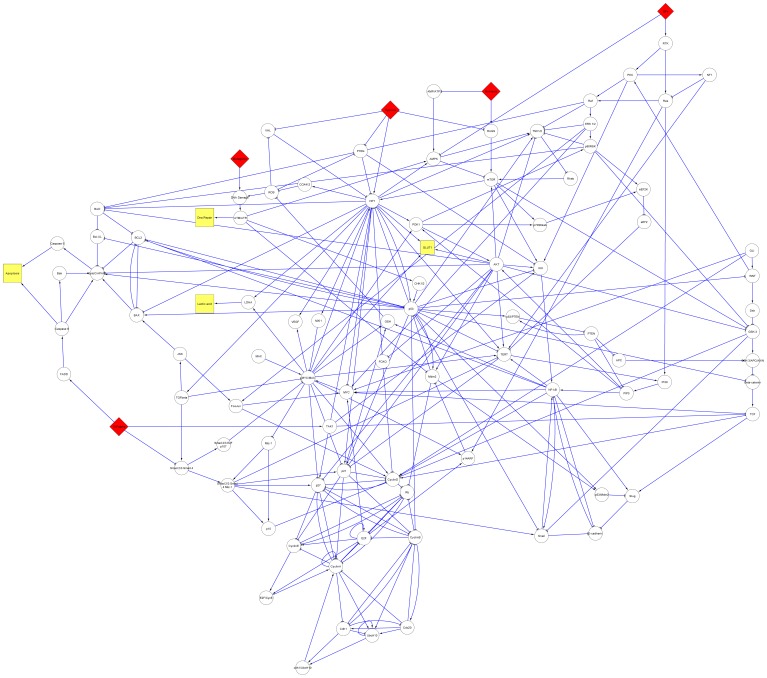
Simplified cancer network. Links correspond to interactions between proteins and to each node is associated a threshold function, eq. 1. Activating interactions are represented by arrows and inhibiting interactions by lines with a bar. The input nodes are shown in red.

The constructed, simplified signaling network is illustrated in Figure 1. It contains 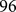 nodes and 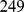 edges. These nodes represent a significant subset of proteins involved in cancer and the network edges produce numerous parallel pathways and alternative routes through which transformed cells can sustain aberrant gene expression patterns, survive and develop further malignancy. The network has 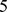 input nodes for applying distinct environmental stimuli and stresses to the cell such as hypoxia, carcinogens, nutrients depletion, proliferative and growth suppressive signalings.

**Figure 2:**
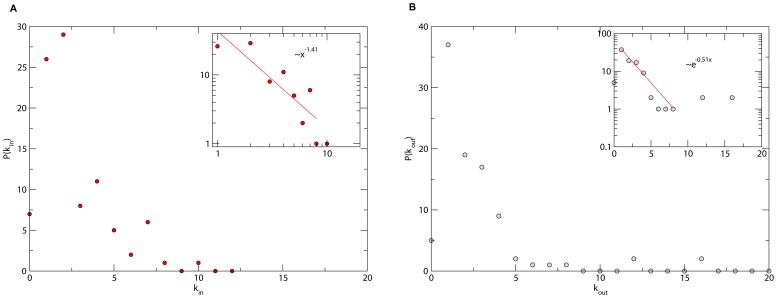
Connectivity distributions for the cancer network. (a) In-degree 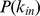 and (b) out-degree 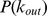 distributions. The insets suggest exponential and power law distributions for the number of nodes regulating and regulated by a given node, respectively.

The topological structure of this cancer network was characterized in terms of its shortest path length, clustering coefficient and connectivity or degree distributions [19]. These quantities were compared with their average counterparts for random networks with the same number of nodes (see Table 1). It can be noticed that the cancer network has a much higher clustering coefficient than random networks. Major features of complex networks are their connectivity distributions. Directed networks are characterized by in-degree 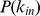 and out-degree 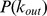 distributions. 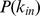 is the probability that a node in the network has 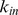 inputs or is “regulated” by other 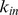 vertices. In turn, 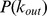 is the probability that a node “regulates” other 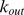 vertices. In Figure 2 these distributions for the cancer network are shown.

**Table 1:** Topological properties of the cancer network and their average counterpats for an ensemble of 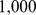 random networks.

Network property	Cancer	Random
nodes	96	96
edges	249	249±12
mean connectivity	2.59	2.59±0.12
shortest path length	3.14	2.91±0.08
clustering coefficient	0.178	0.026±0.005

### A Boolean Dynamics for Cancer Pathways

Each protein *i* , a node in the network, is represented by a binary state 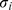, 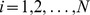. When 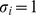 the protein is functionally active. On the contrary, when 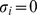 the protein is inactive. The network state at a given time 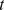 is specified by its protein activity pattern 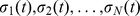. Each protein 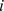 interacts with 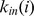 other input proteins. These inputs are all nodes in the network from which a directed link is sent towards the protein 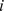, including eventually itself. So, 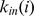 is the in-degree of the node 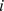. In turn, each link can be either a activating or inactivating interaction. Activation or inhibition can be the result of distinct biochemical mechanisms such as transcriptional regulation, phosphorylation, enzymatic or biding interactions.

The dynamics of the network proceeds in discrete time steps through the simultaneous (parallel) update of the states of its nodes according to the rule.
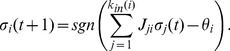
(1)


Here, 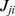 is the interaction strength from input 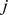 on protein 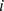. An activation interaction is positive and an inhibitory one is negative. The threshold function 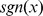 is the unitary step function (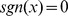 if 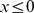 but 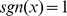 if 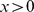). Finally, 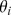 is the activation threshold of protein 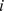. Thus, every protein evaluates the present stimulus from all its inputs. If the overall stimulus it receives at time 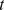 overcomes its activation threshold, the protein activates, or stays active if it was already active; otherwise, it turns inactive or stays inactive. Text S1 lists all the update rules of the model (see Text S1). As emphasized in reference [8], a dramatic simplification inserted in this evolution rule consists in neglect any difference in the time scales of the biochemical interactions involved.

## Results

### The Basic Phenotypes

Since the state space of a Boolean network with 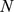 nodes contains 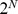 different configurations, its deterministic dynamics, viewed as a flow in this state space, ultimately will drive the system towards attractors. Such attractors are particular subsets of states, either a fixed point, i. e, a single network configuration, or a limit cycle of period 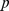, comprised by 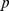 states sequentially visited by the network dynamics. These attractors correspond to specific protein activation patterns and can be interpreted as distinct cell phenotypes.

Thirty two million initial states, associated to all environmental conditions, flowed into 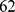 attractors (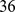 fixed points and 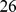 limit cycles). All attractors are listed in the Text S1. The state space is hierarchically organized. At a higher level, it is partitioned into subsets of states by distinct environmental conditions. At a lower level, every of these subsets is subdivided into basins of attraction associated to distinct attractors. None of these attractors can be reached starting from initially distinct environmental conditions. Thus, the repertoire of cell behaviors (attractors) is determined univocally by the cell microenvironment.

**Figure 3:**
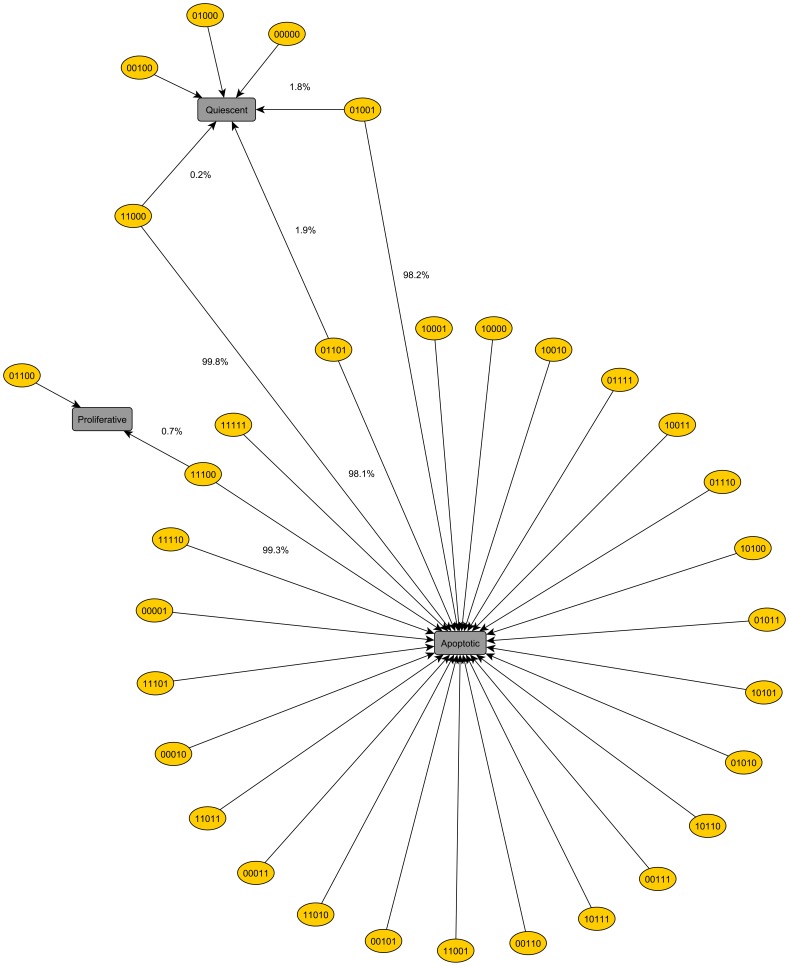
Network's responses to distinct environmental conditions. Three phenotypes (apoptotic, proliferative and quiescent) are generated in response to all 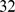 distinct environmental conditions. Here, a microenvironment is specified by the binary sequence of values associated to input nodes (carcinogens, growth factors, nutrient supply, growth suppressors, hypoxia). For instance, the microenvironment (11000) corresponding to a carcinogenic and mitogenic background leads the cell to either an apoptotic (in 99.8% of the initial states) or a quiescent phenotype (rarely, 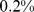). In our network, carcinogens elicit DNA damage and TNF-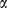 is the suppressive growth signal.

Although distinct, these 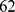 attractors can be classified in groups characterized by specific phenotypes, some of them comprised of very similar elements, i. e, having small Hamming distances among them (Text S1). The phenotypes were defined taking into account the states of a small subset of nodes, instead of all 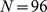 proteins on the network. Considering the effects of mutations, reported on the next subsection, they include the following basic cell phenotypes: apoptotic, characterized by active caspases; glycolytic, with H1f1 activated under normoxia; immortalized, in which hTert is active; migratory, associated to inactivate E-cadherin; mutator, corresponding to inactive Atm/Atr proteins in the presence of DNA damage; proliferative, in which cyclins are activated along the cell cycle in the correct sequence; and quiescent, with cyclins inactive or activated in a wrong sequence. In terms of such phenotypes, the network response to the diversity of microenvironments is highly constrained, as illustrated in Figure 3. Among the 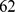 attractors, 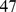 correspond to apoptotic, 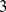 to proliferative and 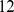 to quiescent phenotypes, which attract 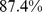, 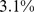, and 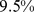 of tested initial states, respectively.

In particular, for some environmental conditions the behaviors emergent from the whole network were compared with those of a normal cell. Under normoxia and adequate nutrient supply, the network reaches from all compatible initial states a fixed point interpreted as a quiescent phenotype. If, in addition to normoxia and nutrient abundance, growth factor signaling is receipt, the network always evolves to a limit cycle associated to a proliferative phenotype. So, as expected, normal cells are totally dependent for their proliferation upon mitogenic signals [20]. Furthermore, under hypoxia, adequate nutrient supply and absence of growth factors, the network is attracted from all compatible initial states to a fixed point corresponding to an apoptotic phenotype. Consistently, programmed cell death is the typical response of somatic cells to many forms of stress such as hypoxia and nutrient deprivation [20].

Under a hypoxic environment with nutrients and growth factors, the network exhibits bistability. It is either attracted to the quiescent phenotype (in 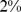 of the initial states) or to the apoptotic phenotype (in 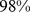 of the initial states). Accordingly, it is known that hypoxia-dependent activation of HIF1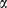 inhibits Myc, leading to cell cycle arrest [21]. Also, HIF1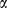 can bind to and stabilize p53, resulting in apoptosis or growth arrest. Bistability is also observed if DNA damage is introduced in a scenario of normoxia, adequate nutrient supply, and mitogenic signaling: around 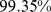 of the compatible initial states are attracted to the apoptotic phenotype, while a very small fraction (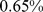) of them reach the proliferative phenotype. Again, it is widely known that the cell cycle is easily interrupted and apoptosis triggered by DNA damage in normal cells. But a proliferative response, although rare, endows altered somatic cells with a proliferative capacity. In our model, this proliferative response is associated to two distinct limit cycles. In one of them the anti-apoptotic signals – Bcl2, Bcl-Xl, and Mdm2 – are consistently active whereas pro-apoptotic signals – Bad, Bax, p53 – are inactive. In the other, Bad and Bax are inactive but p53 and anti-apoptotic signals oscillate in such a way that whenever p53 is activated, the same occurs with Bcl2, Bcl-Xl, and Mdm2. Then, the result is that caspases, the effectors of apoptosis, are always inactive along these limit cycles.

Summarizing, the whole network generates responses coherent with those observed in a normal cell under different somatic environments, indicating the fundamental correctness of the model.

### Mutational Events and Carcinogenesis

We additionally checked the robustness (stability) of attractors to mutations in network nodes and/or links. This is a central feature because incipient cancer cells need to acquire hallmark traits to ultimately become malignant [15] and genome instability underlies these acquisitions. Once a mutation was introduced, the node DNA damage is permanently turned on, activating the Atm/Atr pathway. We focused on the attractors associated to two environmental conditions, namely, adequate nutrient supply and either normoxia or hypoxia, frequently present in early carcinogenesis.

Under normoxia and adequate nutrient supply, it was found that mutations in 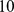 proteins transform the formerly quiescent, normal cell into a proliferating one. These proteins, as well as the nature of the driver mutations and their efficacy are listed in Table 2. In turn, under hypoxia and adequate nutrient supply, mutations in 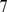 proteins enable the transformed cell to evade apoptosis formerly induced by hypoxia (Table 3). The protein Nf-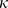
B is common to Tables 2 and 3, hence it can enable a transformed cell to simultaneously acquire proliferative capacity and evading apoptosis.

**Table 2:** Driver mutations under normoxia.

Protein	mutation	efficacy
Egfr	activation	0.91%
	overexpression	0.91%
Gli	activation	0.08%
	overexpression	0.08%
hTert	activation	0.08%
	overexpression	0.07%
Nf1	deletion	0.03%
Nf-*κ*B	overexpression	0.13%
Pi3k	activation	0.14%
	overexpression	0.73%
Pkc	activation	25%
	overexpression	0.73%
Pten	deletion	0.51%
Ras	activation	0.16%
Wnt	activation	0.6%
	overexpression	0.6%

Targeted proteins and corresponding mutations that drive the network into a proliferative phenotype under normoxia and adequate nutrient supply. The efficacy of a mutation was defined as the fraction of initial states that are driven to the new phenotype.

**Table 3:** Driver mutations under hypoxia.

Protein	mutation	efficacy
Akt	overexpression	100%
Bcl2	activation	100%
	overexpression	100%
Bcl-Xl	overexpression	100%
Ikk	overexpression	88.7%
Nf-*κ*B	activation	91.7%
	overexpression	100%
p53	deletion	100%
Snail	overexpression	83.6%

Targeted proteins and corresponding mutations that enable the network to evade apoptosis induced by hypoxia. The efficacy of a mutation was defined as the fraction of initial states that are driven to the new phenotype.

We also investigated the effect of defective DNA integrity sensors that impair a cell to detect the occurrence of mutations. Now, the node DNA damage is permanently turned off and does not activates the Atm/Atr pathway. Under normoxia and adequate nutrient supply, the number of mutated proteins that transform a quiescent, defective cell into a proliferating one increases to 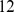. Yet, the number of mutated proteins that confer to a hypoxic, defective cell the capacity to evade apoptosis also increases to 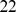. These proteins include Akt, Bcl2, Egfr, Nf-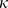
B, p53, Pi3k, Pten, Ras, and Wnt (see Tables S3 and S4). In the absence of an intact DNA damage repair pathway, in which Atm and Atr play central roles, our results indicate that network attractors become more prone to structural changes or, in biological terms, exhibit increased genomic instability.

Finally, we investigated if nodes whose mutations can confer hallmark capabilities to the transformed cell have special status in network topology. For all them their betweenness centrality 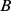 were determined [19]. Three groups have been observed. The first one, comprised of eight nodes (Akt, Hif1, hTert, Ikk, mTor, Myc, Nf-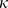
B, and p53), has more than twice the network average centrality 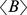. Further, the group average connectivity is 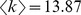 and seven of its elements are highly connected (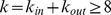). These results indicate the centrality of the nodes in this group for which converge or through which crosstalk distinct signaling pathways. The second group, containing nodes with 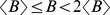, includes Mdm2 and Pdk1. In addition, the group average connectivity is 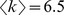 and half of them exhibits intermediate connectivities (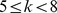). At last, the third group is characterized by small 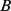 (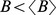) and connectivities (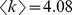). Among its elements, Bcl2,Bcl-x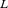, Egfr, Gli, Nf1, Phd, Pi3k, Pkc, Pten, Ras, Snail, Vhl, and Wnt, nine have small connectivities 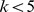. Even though these nodes are not topologically central, almost all are nearest neighbors (direct regulators) of central nodes from the first group. Hence, they assume major roles in network dynamics.

#### Colorectal carcinogenesis

Here, we investigate if cancer cells need only a few driver mutations (those that change phenotypic traits) to deal with all environmental constraints and advance towards a fully malignant phenotype. As a paradigm, the carcinogenesis of the colorectal cancer was considered. So, the first mutations introduced in the network were Apc deletion and Tcf interactions with their targets strengthened by a factor 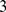. These mutations lead to a structural instability of the network “phase portrait”. Now, there are 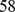 attractors, 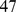 apoptotic, 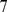 proliferatives, and 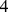 quiescent, which attract 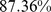, 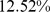 and 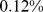 of tested initial states, respectively. The number of proliferative attractors and the sizes of their basins increased at the expenses of the quiescent attractors. However, no anti-apoptotic advantage was observed. As expected, the network response was environmental dependent. Under normoxia and nutrient availability, these mutations lead to a proliferative phenotype. However, this proliferative advantage is lost under hypoxia or genotoxicity (DNA damage), when apoptotic phenotypes are observed.

Next in the sequence, a new mutation – Ras constitutively activated – was implemented. As a result the network dynamics exhibits 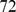 attractors, 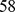 apoptotic, 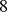 proliferatives, and 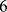 quiescent, which attract essentially the same fractions of tested initial states (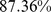, 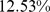 and 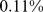, respectively) as before (Apc and Tcf mutated). However, this additional mutation conferred to the network a small chance to overcome the hypoxic barrier, sustaining quiescent phenotypes for 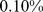 of tested initial states under a hypoxic, but nutrient rich environment. Further, constitutive Ras activation also can lead to a proliferative phenotype in 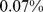 of initial states in a normoxic, nutrient rich, but genotoxic environment.

In the sequence, Smad4 was deleted. This mutation increases to 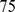 the number of attractors, 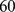 apoptotic, 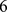 proliferative, and 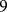 quiescent. The network acquires proliferative phenotypes for all initial states under normoxia and adequate nutrient supply, even receiving inhibitory growth signaling provided by a constitutively active Tgf-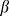. It is worthy to mention that the network exhibits only apoptotic and quiescent attractors if Tgf-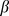 is constitutively active and Smad4 is functional (undeleted). So, Smad4 deletion in accumulation with the previous mutations endows the transformed network with the capacity to evade suppressive growth signals.

The next mutations were Pten deletion and doubling of Akt interaction strengths. In consequence, the number of attractors further increases to 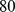, 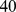 apoptotic, 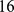 proliferative, and 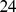 quiescent, which attract 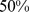, 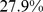 and 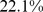 of tested initial states, respectively. Again, different microenvironments elicit distinct responses. Under normoxia and adequate nutrient supply the network always exhibit aggressive (proliferative, glycolitic and immortalized) phenotypes. But if hypoxia replaces normoxia, in addition to proliferative, glycolitic and immortalized phenotypes which attract 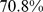 of the initial states, there are quiescent attractors toward which 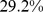 of initial states converge. Adding growth suppressors or DNA damage to the former microenvironment can at most lead to quiescence. For instance, in normoxic, nutrient rich and genotoxic microenvironment, 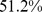 of initial states are driven to proliferative, glycolytic and immortalized attractors, whereas 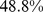 of them are driven to quiescent attractors. Therefore, since hypoxia or functional DNA damage sensors can lead to quiescent phenotypes, some constraints persist impairing tumor growth.

The last mutation was p53 deletion. Its result is decrease to 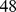 the number of attractors, 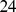 apoptotic and 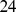 proliferative, both attracting 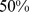 of the initial states. Indeed, apoptosis for 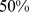 of the initial states is the minimum value possible because in our network active TNF-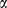 leads to p53-independent activation of caspases. Nevertheless, the main result is that the network always exhibits proliferative, glycolytic and immortalized phenotypes in microenvironments with adequate nutrient supply, hypoxic or normoxic, even genotoxic, which activate DNA damage sensors, and under growth suppressor signaling. Almost all barriers to tumor growth were overcome after this sequence of few mutations.

**Figure 4:**
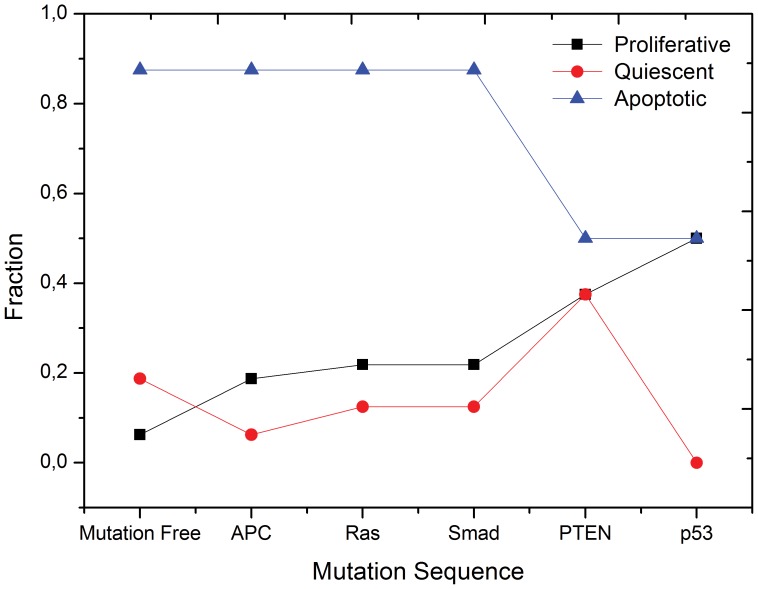
Network response to driver mutations in colorectal carcinogenesis. Fraction of initial states evolving into apoptotic, proliferative or quiescent attractors (phenotypes) for all environmental conditions after the sequential accumulation of each driver mutation in colorectal cancer.

In summary, as shown in Figure 4, our simulations reveal that each driver mutation in the canonical route for the colorectal cancer [22] contributes to increase either the proliferative capacity or the resistance to apoptosis of the transformed cell. In particular, although Smad4 is mutated in only 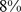 of colorectal cancers, its mutation in concert with the others in the classical colorectal carcinogenesis model generates more aggressive tumor cells. Indeed, their associated proliferative phenotypes attract 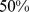 of the initial states against only 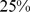 in the absence of the Smad4 mutation. Further, the model indicates that other mutations outside this classical route of colorectal carcinogenesis also leads to proliferative and apoptotic resistant phenotypes. These are the cases, for instance, of Pten, or p53, or Atm, or Fadd, or Chk deletions after Apc and Ras mutations. Alternatively, the constitutive activation of Pi3k, or Akt, or Bcl2, or Mdm2 again after Apc and Ras mutations decreases apoptosis and increases proliferation.

### The Outcomes of Targeted Therapies

The rationale of targeted therapy is inhibit critical, functional nodes in the oncogenic network to elicit the cessation of the tumorigenic state through apoptosis, necrosis, senescence, or differentiation [23]. We performed a survey of nodes in our Boolean model whose inhibition or activation (reintroduction of wild-type proteins) either increase the basins of attraction of apoptotic and quiescent phenotypes or decrease those associated to proliferative phenotypes. Specifically, as a model for fully developed colorectal cancer cells, a network carrying mutations in Apc, Ras, Smad4, Pten, and p53, was considered. For this network, only two phenotypes were observed under all microenvironmental conditions and from every initial state tested: apoptotic (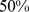) and proliferative (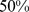). All nodes were individually targeted. Our simulations reveal that the inhibition of Pdk1, Akt, E2F, Cyclins D and E, and Mdm2 enhances quiescent phenotypes (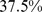 for Pdk1 and Akt, and 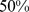 for E2f, cyclins and Mdm2) and also impair the proliferative ones (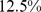 for Pdk1 and Akt, and 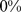 for E2f, cyclins and Mdm2). The same is observed with the activation of p53, Rb, Cdh1, p21, p27, and Smad4 (quiescence increases to 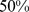 and proliferation vanishes for p53, Rb, Cdh1, p21 and p27, whereas for Smad4 both fractions changes to 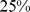). In turn, the activation of FADD and Caspases enhances apoptosis to 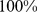 of chance. Paradoxically, the model predicts that either inhibition or activation of Cyclins A and B as well as Cdc20 enhances quiescence and impair proliferation (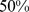 and 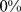, respectively).

Concerning the combinatorial application of drugs currently under clinical trial [23], the effects of several targeted inhibitors on our colorectal cancer cell model were investigated under three environmental conditions, namely, normoxia and adequate nutrient supply, hypoxia and adequate nutrient supply, and normoxia, adequate nutrient supply and carcinogenic stress. For these conditions our colorectal cancer cell model is always attracted to a proliferative, immortalized and glycolytic phenotype. According our simulations the combined use of mTor and hTert inhibitors reverses two malignant hallmarks: glycolysis and immortalization; the association of mTor and Cdks inhibitors prevents glycolysis and cell cycling; and the combination of Cdks and hTert inhibitors prevents proliferation and immortalization. So, since in our model monotherapies with mTor, Cdks, and hTert inhibitors prevents glycolysis, cell division, and immortalization, respectively, the therapeutic outcome seems to be additive or “linear” (i. e., obeys a superposition principle). In turn, the association of p53 reactivators and Bcl-2/Bcl-Xl inhibitors leads to cell cycle arrest under hypoxia and adequate nutrient supply or normoxia, adequate nutrient supply and carcinogenic stress, but has no effect under normoxic and nutrient rich microenvironments. Another association involving p53 reactivators and Vegf inhibitors blocks cell cycling in 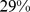 of the initial states tested in these three environmental conditions. In our model, the action of Vegf inhibitors is simulated by forcing hypoxia and nutrient depletion on the input nodes.

Finally, we tested mono- and two-drugs therapies using mTor, Vegf, Ras, and Pkc inhibitors in cells at distinct stages of malignancy. Again, our colorectal cancer cell model was considered. Our results reveal that a full malignant cell (network carrying mutations in Apc, Ras, Smad4, Pten, and p53) at most lost its glycolytic phenotype if mTor is inhibited by a therapeutic agent, but its limitless proliferative capacity remains unchanged. In contrast, a cell in the previous carcinogenic stage (network with mutations in Apc, Ras, Smad4, and Pten) can have its chance of proliferation decreased under both monotherapy and two-drugs association. Indeed, the fraction of proliferative phenotypes decreases from 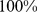 to about 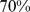, 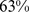, and 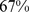 using Vegf, mTor 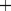 Vegf, and Pkc 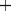 Vegf inhibitors, respectively. Also, the therapeutic outcome depends on the external environment. For instance, combined mTor and Pkc inhibition reduces the proliferation chance from about 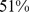 to 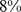 under normoxia, adequate nutrient supply and carcinogenic stress, but this change is significantly smaller under hypoxia and adequate nutrient supply, from about 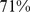 to 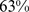. So, these results demonstrate that each treatment distinctly affects cells in different grades of malignancy and eventually clones will emerge, rendering the therapy ineffective.

## Discussion

We constructed a Boolean dynamical system integrating the main cancer signaling pathways in a simplified network. The dynamics of this network is controlled by attractors associated to apoptotic, proliferative and quiescent phenotypes that qualitatively reproduce the behaviors of a normal cell under diverse microenvironmental conditions. Indeed, the network response is highly constrained with 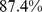, 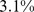, and 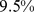 of the initial states attracted to apoptotic, proliferative and quiescent phenotypes, respectively. So, under persistent stress, apoptosis or cell cycle arrest are the rule. Further, cell proliferation is tightly regulated, occurring almost only in a normoxic environment and in the presence of growth signaling. As observed in our model, GF signaling significantly increases the stability of the surviving (proliferative and quiescent) phenotypes while inhibits apoptosis. This result is consistent with the findings of Mai and Lieu [13] that, using a Boolean network integrating both the intrinsic and extrinsic pro-apoptotic pathways with pro-survival GF signaling, demonstrated that apoptosis can be induced either easily or difficultly depending on the balance between the strengths of pro-apoptotic and pro-surviving signals.

Our simulational results demonstrate that perturbations in some network nodes elicit phenotypic transitions. We interpreted them as driver mutations and can represent either the constitutive activation or inactivation of a node or yet an increase in the interaction strengths of a node with its targets. Under normoxia and adequate nutrient supply, we found that mutations in Egfr, Gli, Nf1, Nf-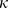
B, Pi3k, Pkc, Pten, Ras, and Wnt transform the formerly quiescent, normal cell into a proliferating one. The resultant clonal expansion often leads to hypoxia. Additional mutations in Akt, Bcl2, Bcl-Xl, Ikk, Nf-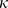
B, p53 and Snail enable the transformed cell to evade apoptosis formerly induced by hypoxia. These 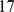 driver mutations predict by our model are included among the approximately 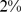 of genes in the human genome causally implicated in tumor progression by diverse census of cancer genes recently performed [24], [25], [26]. The predicted drivers clusters on certain signaling pathways as, for instance, in the classical Mapk/Erk (Egfr, Nf1 and Ras), Pi3k (Pi3k, Pkc, Pten, Akt), p53 and Wnt signaling pathways. Also, sequencing data reveal that some of them are significantly mutated in cancers: Pi3k, Pten, and Akt in breast cancer [26], [27]; Ras and p53 in either breast and colorectal cancers [26], [28]; p53 and Nf1 in ovarian carcinoma [29]; p53 and Pten in small-cell lung cancer [30]; and Egfr, p53, Nf1, and Pi3k in human glioblastoma [31]. In turn, Gli is target of translocation or amplification in either breast and colorectal cancers [28], Bcl2 is amplified in squamous cell lung cancers [32] and the Wnt signaling pathway is altered in 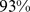 of all colon and rectal cancer [33]. The other predicted drivers realize a main result obtained from our model: driver nodes are not necessarily central in the network topology, but at least they are direct regulators of central components towards which converge or through which crosstalk distinct cancer signaling pathways.

Our Boolean model for cancer pathways allowed us to explore carcinogenesis at the molecular level. Carcinogenesis is an evolutionary process driven by a sequential acquisition of stepwise, somatic-cell mutations with concomitant subclonal selection [34]. Cells in a nascent tumor are continuously facing environmental stresses often in response to inflammation. Inflammation elicits cytokine-induced hyperplasia, genotoxicity, and ROS-induced cell death. In hyperplasic epithelia, abnormal cell growth leads first to hypoxia and selection for a glycolytic phenotype, resulting in increased acidity and nutrient and growth factor deprivation. Severe chronic hypoxia can select for apoptosis resistance or mutated p53, further promoting the accumulation of mutations. Low pH can generate DNA damage and glucose deprivation strongly reinforces the selection for activated oncogenes. The spatial heterogeneity of microenvironments within a primary tumor selects for cell migration, which may produce invasion and metastasis. So, as was demonstrated by our simulations of the colorectal carcinogenesis, in order to evolve, cancer cells perform sequential and random searches for phenotypic solutions to overcome the barriers imposed by their altering environment. Ultimately, each driver mutation in a carcinogenic route contributes to increase either the proliferative capacity or the resistance to apoptosis of the transformed cell, while several “passenger” mutations can accumulate along the process. Our Boolean model supports distinct carcinogenic routes characterized by specific sets of critical mutations embedded within varied spectra of passenger mutations. Thus, as observed in genomic analysis of diverse cancers, different combinations of mutated genes exist and most samples of a given tumor type differ from all others [26].

The development of effective targeted therapies copes with a major challenge. From the experimental view point it is very hard to determine which proteins have critical roles in tumor progression to be targeted pharmacologically. This is where mathematical models integrating diverse cancer pathways can be helpfull. Using our model, we find that the inhibition of some nodes (Pdk1, Akt, E2F, Cyclins D and E, and Mdm2) or the activation of others (p53, Rb, Cdh1, p21, p27, and Smad4) enhances quiescent phenotypes and also impairs the proliferative ones. However, all the monotherapies tested were ineffective to simultaneously reverse all the malignant hallmarks and seems to be additive or “linear” in their effects. Thus, our simulations indicate that the association of targeted drugs is necessary for cancer control or eradication. Further, we find that each treatment distinctly affects cells in different grades of malignancy or subject to distinct microenvironmental conditions. Although a combined targeted therapy can eradicate a set of cancer cells with certain phenotypes, eventually clones will emerge, rendering the therapy ineffective. So, these results provide additional support to the view that a combinatorial series of drugs applied concurrently to block cancer pathways and alter the tumor environment is needed to eradicate all of the cancer cells in a tumor [23]. However, in contrast to the present model focusing an isolated cell, tumors are highly heterogeneous systems on larger length scales patterned in patches (local microenvironments) that continuously change in space and time. Thus, a single drug association can have a limited effectiveness or to be efficacious demands a large number of therapeutic agents which imposes unacceptable clinical risks.

Finally, we briefly comment on the limitations and prospects of our modeling approach. In its present form, the cancer network and its Boolean dynamics are able to reproduce some biologically relevant features of carcinogenesis. It will certainly be true that further improvements on the topology of the cancer network will lead to better results and predictions. Indeed, adding new nodes, links and signaling pathways to the network can generate new convergences and redundancies essential to achieve more accurate predictions concerning cancer gene candidates and effective targets for therapies. Despite these rather straightforward secondary advances, the primary limitation of the model lies in its own single-scale nature constrained to the molecular level of a cell. Ultimately, cell-cell interactions in a changing environment determine which of the possible transformed cell clones will be generated and selected along cancer progression and, in consequence, the efficacy and safety of molecularly targeted therapies. Hence, Boolean network models for cancer pathways might be suitable to describe the microscopic scale in multiscale models of cancer growth and therapy [6]. Currently, we are pursuing this goal by extending our previous multiscale models considered in references [35] and [36].

## Materials and Methods

The network integrating the main signaling pathways involved in cancer was constructed based on the current literature and protein-protein interactions map reported on KEGG database [1], [16], [37]. Specifically, subgraphs of the PI3K-AKT, mTOR, MAPK, HIF1, TGF-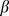, WNT, NF-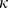
B, TNF, cell cycle, p53, and apoptosis KEGG pathways were included in our network model. Detailed descriptions of these pathways can be found in several comprehensive reviews [38], [39], [40], [18], [41], [42], [43], [44], [45], [46]. Its input nodes, i. e., those which are not regulated by other nodes and therefore associated to environmental cues, represent growth factors, nutrient and oxygen supplies, carcinogens (or mutagens), and apoptotic signal (Tnf-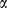). This simplified network focuses attention on those gene products and signaling pathways that are implicated more universally in cancer and which seems to be sufficient to generate a simple model still capable of explaining some general features of carcinogenesis.

Almost all activating interactions have a strength 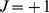, whereas the inhibitory ones have 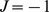. The exceptions are listed in Table 4 and totalizes three of the 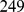 network links. Similarly, the majority of the activation thresholds has 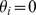, but there are 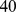 nodes among the 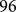 proteins with different values. They are also listed in Table 4. The assignment of each threshold value was guided by the local network topology (the inputs of each node) and very general dynamical arguments or expected biological responses of normal cells (see comments in Table 4). For instance, some nodes are “regulated” only by inhibitory inputs. For them, 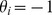 in order to ensure their activation and the spreading of their signals on the network if those inputs are inactive. Other nodes are binary protein complexes whose activation thresholds were set to 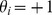 because their two constituents must be activated to form the complexes.

**Table 4:** Interaction strengths and activation thresholds with special values.

Strength	Nature	Protein interaction
+2	Activation	Nf-*κ*B → Bcl-2
		Ikk → Nf-*κ*B
−2	Inactivation	Gsk-3 → Cyclin D
		Rb → E2f
		Vhl → Hif1
Threshold	Protein	Comment
−3	Gsk-3	Active in the absence of GFs.
	E-cadherin	Active in non-transformed epithelial cells.
	Rb	Active in non-cycling cells.
−2	Foxo	Active unless Akt is superexpressed.
	Hif1	Active under hypoxia.
	Max	Node without inputs; so it is constitutively activated.
	Ras	Activated by GFs or Nf1 inactivation.
	E2f	Activated by GFs
	p21	Active in non-transformed cells.
	p53	Inactive in NTU cells.
	Ampk	
	Mdm2	Active in NTU cells.
	Phds	
−1	Vhl	
	p27	Active in non-cycling cells in normoxia and GFs free.
	Apc	Constitutively activated.
	Nf1	Active in non-transformed, non-cycling cells.
	Pip3	Activated by GFs.
	Tsc1/2	Active in NTU cells free from GFs.
	Cyclin B, Rheb	Only inhibitory inputs. The node is activated if its
	*β*-catenin, Cdh1	inhibitors are inactive.
	eEf2, Miz-1, Pten	
	Bad, Bcl-Xl, AMP/ATP	
	p53/Pten, Myc/Max,	Binary complexes formed only if its component
	Gsk-3/Apc, E2f/Cyclin E,	parts are activated.
	Cdh1/UbcH10, Smad/Miz-1,	
	p53/Mdm2	
	Akt	Demand both of its inputs activated.
+1	mTor	Active in proliferating, NTU cells.
	Glut1	Inactive in normoxia and proliferative signals absent.
	Nf-*κ*B	Inactive in NTU cells free from GFs.
	Myc	Inactive in NTU cells free from GFs.
	Ldha	Inactive in normoxic, non-transformed cells.
	Snail	Ativated by TGF-*β* and proliferative signals.
+3	p14*^ARF^*	Active under E2f overexpression.
+4	HTert	Inactive in non-immortalized cells.

Abbreviations: GFs, growth factors; NTU cells, non-transformed, unstressed (normoxia, adequate nutrient supply, undamaged DNA, mutation free, etc.), non-proliferating cells.

In our simulations, every environmental condition, each one corresponding to a fixed set of input nodes, were analyzed individually. Fixed the input nodes, 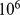 initial states for the other nodes were randomly chosen according the following protocol. Firstly, 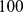 distinct and equally spaced values for the probability 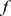 of a node to be active, 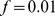, 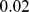, 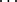, 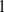, were used. Secondly, 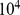 configurations for the non-input nodes were randomly generated for each value of 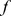.

In order to analyze the stability of the network attractors, two types of mutations were implemented: constitutive activation or inactivation of a chosen protein 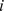 (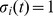 or 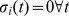, respectively), and protein overexpression (constitutive activation with concomitant increasing in its interaction strengths 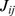 with its target proteins). In the simulations, all nodes were subjected to such mutations and their effects were observed for 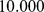 randomly chosen initial states.

The simulations were performed in gfortran using a microcomputer with a Intel i3-2310 M, 2.1 GHz dual core processor and 4 Gb RAM. The network was drawn with Cytoscape [47].

## Supporting Information

Figure S1
**Attractors observed under distinct microenvironmental conditions.** These microenvironments are described by their binary codes listed at the left margin of the figure. Specifically, 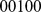 (normoxic and nutrient rich), 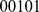 (normoxic and plenty of nutrients and growth factors), 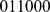 (hypoxic and nutrient rich), 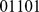 (hypoxic, plenty of nutrients and growth factors), and 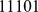 (normoxic, plenty of nutrients and growth factors, and under genotoxic stress – carcinogens). These conditions frequently occur either during different stages of carcinogenesis or in certain regions within spatially heterogeneous solid tumors.(PDF)

Table S1
**Fixed points of the cancer network.** Fixed points for all 32 possible environmental conditions.The basin size of a fixed point was estimated as the fraction of 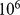 initial states driven to that attractor.(PDF)

Table S2
**Limit cycles of the cancer network.** Limit cycles for all 32 possible environmental conditions. The basin size of a cycle was estimated as the fraction of 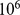 initial states driven to that attractor.(PDF)

Table S3 **Driver mutations under normoxia.** New driver mutations under normoxia and adequate nutrient supply in the context of defective DNA integrity sensors.
(PDF)

Table S4 **Driver mutations under hypoxia.** New driver mutations under hypoxia and adequate nutrient supply in the context of defective DNA integrity sensors.
(PDF)

Text S1 **Supporting information text file.**
(PDF)
