# Supplementary figures and images for: Boolean Network Model for Cancer Pathways: Predicting Carcinogenesis and Targeted Therapy Outcomes
Source: PLoS One. 2013 Jul 26;8(7):e69008. doi: 10.1371/journal.pone.0069008 (PMC3724878; doi:10.1371/journal.pone.0069008)

☐ Inactive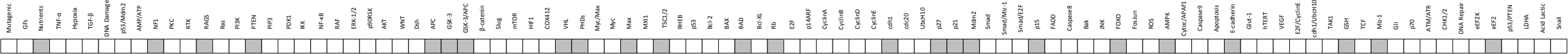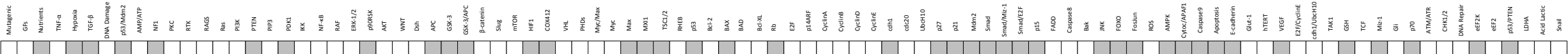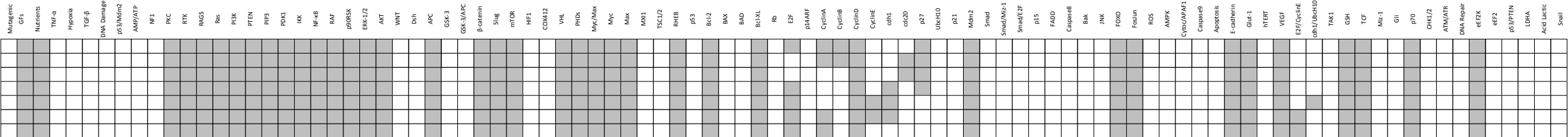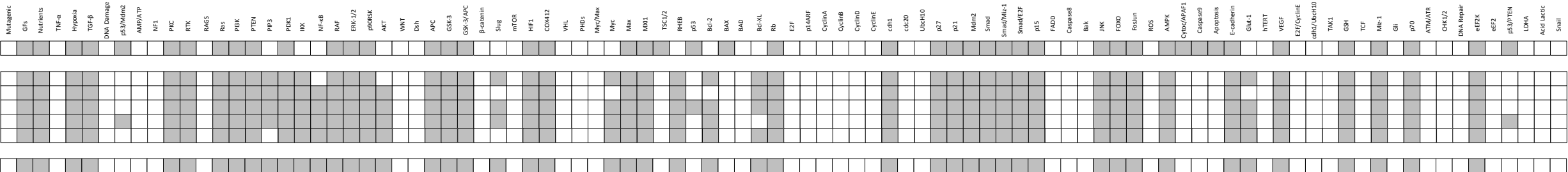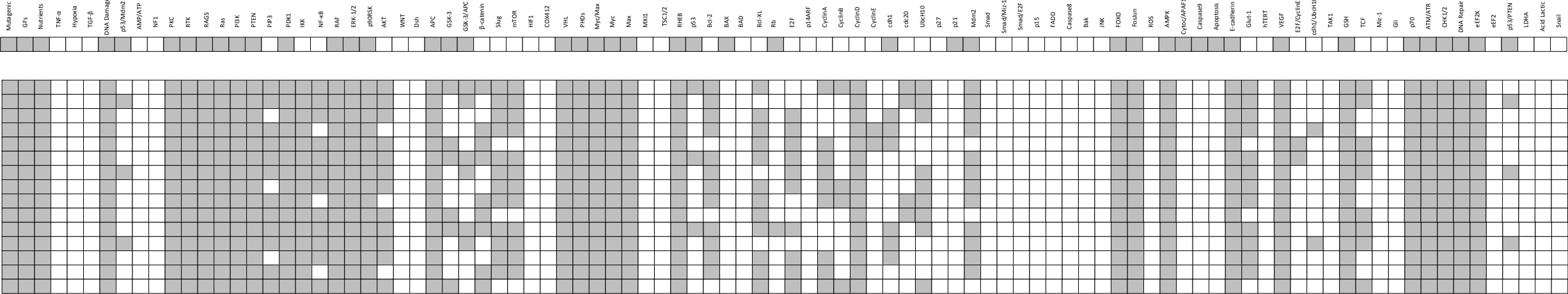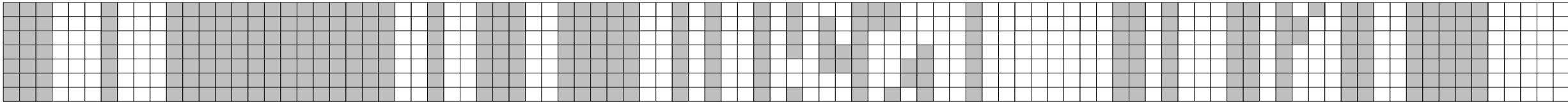

Supplement: Figure S1 — Attractors observed under distinct microenvironmental conditions. These microenvironments are described by their binary codes listed at the left margin of the figure. Specifically, (normoxic and nutrient rich), (normoxic and plenty of nutrients and growth factors), (hypoxic and nutrient rich), (hypoxic, plenty of nutrients and growth factors), and (normoxic, plenty of nutrients and growth factors, and under genotoxic stress – carcinogens). These conditions frequently occur either during different stages of carcinogenesis or in certain regions within spatially heterogeneous solid tumors. (PDF) [file pone.0069008.s001.pdf]
