# Supplementary material for: Boolean Network Model for Cancer Pathways: Predicting Carcinogenesis and Targeted Therapy Outcomes
Source: PLoS One. 2013 Jul 26;8(7):e69008. doi: 10.1371/journal.pone.0069008 (PMC3724878; doi:10.1371/journal.pone.0069008)
Supplement: Table S1 — Fixed points of the cancer network. Fixed points for all 32 possible environmental conditions.The basin size of a fixed point was estimated as the fraction of initial states driven to that attractor. (PDF) [file pone.0069008.s002.pdf]

Environmental conditions	Attractors		
	number	basin size	phenotype
00000	1	1	quiescent
00001	1	1	apoptotic
00010	1	1	apoptotic
00011	1	1	apoptotic
00100	1	1	quiescent
00101	1	1	apoptotic
00110	1	1	apoptotic
00111	1	1	apoptotic
01000	1	0.9846	quiescent
01001	2	0.9807	apoptotic
		0.0034	quiescent
01010	1	0.9687	apoptotic
01011	2	1	apoptotic
		0.0015	apoptotic
01100	-	-	-
01101	2	0.9807	apoptotic
		0.0038	quiescent
01110	-	-	-
01111	2	0.8096	apoptotic
		0.0015	apoptotic
10000	1	1	apoptotic
10001	1	1	apoptotic
10010	1	1	apoptotic
10011	1	1	apoptotic
10100	1	1	apoptotic
10101	1	1	apoptotic
10110	1	1	apoptotic
10111	1	1	apoptotic
11000	2	0.9983	apoptotic
		0.0013	quiescent
11001	1	1	apoptotic
11010	2	0.9795	apoptotic
		0.0019	apoptotic
11011	1	1	apoptotic
11100	1	0.9935	apoptotic
11101	1	1	apoptotic
11110	1	0.8827	apoptotic
11111	1	1	apoptotic
