# Supplementary material for: Boolean Network Model for Cancer Pathways: Predicting Carcinogenesis and Targeted Therapy Outcomes
Source: PLoS One. 2013 Jul 26;8(7):e69008. doi: 10.1371/journal.pone.0069008 (PMC3724878; doi:10.1371/journal.pone.0069008)
Supplement: Table S2 — Limit cycles of the cancer network. Limit cycles for all 32 possible environmental conditions. The basin size of a cycle was estimated as the fraction of initial states driven to that attractor. (PDF) [file pone.0069008.s003.pdf]

Environmental conditions	Attractors			
	number	period	basin size	phenotype
01000	2	2	0.0109	quiescent
		2	0.0045	quiescent
01001	2	2	0.0224	apoptotic
		2	0.0089	apoptotic
01100	1	7	1	proliferative
01101	1	5	0.0155	quiescent
01110	1	7	1	apoptotic
01111	3	5	0.1403	apoptotic
		5	0.0313	apoptotic
		5	0.0173	apoptotic
11000	2	2	0.0002	quiescent
		5	0.0002	quiescent
11010	4	5	0.0153	apoptotic
		10	0.0026	apoptotic
		2	0.0004	apoptotic
		2	0.0002	apoptotic
11110	4	5	0.8682	apoptotic
		25	0.0186	apoptotic
		5	0.0093	apoptotic
		7	0.0026	apoptotic
