# Supplementary material for: Boolean Network Model for Cancer Pathways: Predicting Carcinogenesis and Targeted Therapy Outcomes
Source: PLoS One. 2013 Jul 26;8(7):e69008. doi: 10.1371/journal.pone.0069008 (PMC3724878; doi:10.1371/journal.pone.0069008)
Supplement: Table S3 — Driver mutations under normoxia. New driver mutations under normoxia and adequate nutrient supply in the context of defective DNA integrity sensors. (PDF) [file pone.0069008.s004.pdf]

Protein	mutation	efficacy
Akt	overexpression	54.3%
Egfr	activation	100%
	overexpression	100%
Gli	activation	100%
	overexpression	100%
hTert	activation	100%
	overexpression	100%
Nf1	deletion	100%
Nf-κB	overexpression	2.1%
Pdk1	overexpression	36%
Pi3k	activation	100%
	overexpression	100%
Pkc	activation	100%
	overexpression	100%
Pten	deletion	100%
Ras	activation	100%
	overexpression	100%
Wnt	activation	100%
	overexpression	100%
