# Supplementary material for: Boolean Network Model for Cancer Pathways: Predicting Carcinogenesis and Targeted Therapy Outcomes
Source: PLoS One. 2013 Jul 26;8(7):e69008. doi: 10.1371/journal.pone.0069008 (PMC3724878; doi:10.1371/journal.pone.0069008)
Supplement: Table S4 — Driver mutations under hypoxia. New driver mutations under hypoxia and adequate nutrient supply in the context of defective DNA integrity sensors. (PDF) [file pone.0069008.s005.pdf]

Protein	mutation	efficacy
Akt	overexpression	100%
Bcl2	activation	100%
	overexpression	100%
Bcl-x_L	overexpression	100%
Egfr	activation	2.6%
	overexpression	2.6%
Gli	activation	0.7%
	overexpression	3.05%
Hif1	deletion	100%
hTert	activation	0.09%
	overexpression	0.09%
Ikk	overexpression	99%
Mdm2	overexpression	100%
mTor	overexpression	19%
Myc	activation	0.03%
	overexpression	0.02%
Nf1	deletion	0.08%
Nfκ-B	activation	99%
	overexpression	100%
p53	deletion	100%
Phd	overexpression	100%
Pkc	activation	2.6%
	overexpression	9.1%
Pi3k	activation	0.18%
	overexpression	17.3%
Pten	deletion	14.8%
Ras	activation	0.17%
	overexpression	0.17%
Snail	overexpression	98%
Vhl	activation	100%
	overexpression	100%
Wnt	activation	3.5%
	overexpression	3.5%
