# Supplementary material for: Boolean Network Model for Cancer Pathways: Predicting Carcinogenesis and Targeted Therapy Outcomes
Source: PLoS One. 2013 Jul 26;8(7):e69008. doi: 10.1371/journal.pone.0069008 (PMC3724878; doi:10.1371/journal.pone.0069008)
Supplement: Text S1 — Supporting information text file. (PDF) [file pone.0069008.s006.pdf]

Supporting information — Boolean Network Model for Cancer Pathways: Predicting Carcinogenesis and Targeted Therapy Outcomes.

Herman F. Fumiã, Marcelo L. Martins[1,*]
Departamento de Física, Universidade Federal de Viçosa, Viçosa, Minas Gerais, Brazil
[1] National Institute of Science and Technology for Complex Systems, Brazil
[*] E-mail: mmartins@ufv.br

Evolution rules

The cancer network proposed, although highly simplified, includes proteins associated to oncogenes, tumor-suppressor genes and stability genes, the three major classes of targets for the mutations involved in tumorigenesis. These targets act through a relatively small number of signal transduction pathways that regulate cell replication, apoptosis, and DNA damage response. Specifically, our network model considers subsets of the PI3K-AKT, mTOR, MAPK, HIF1, TGF-β, NF-κB, TNF, WNT, Rb-E2F, p53, and apoptosis pathways. Many cancer-associated proteins, such as p53, Nf-κB, Egfr, Ras, Myc, Pten, Hif1, and Bcl2 are involved in more than one pathway and there is substantial cross-talk between pathways.

The update rules for each one of the 96 nodes on the network follow:

$$\sigma_{Mutagen}(t+1) = input;$$

$$\sigma_{GFs}(t+1) = input;$$

$$\sigma_{Nutrients}(t+1) = input;$$

$$\sigma_{TNF\alpha}(t+1) = input;$$

$$\sigma_{Hypoxia}(t+1) = input;$$

$$\sigma_{Gli}(t+1) = input;$$

$$\sigma_{TGF\beta}(t+1) = sgn[+\sigma_{HIF1}(t)];$$

$$\sigma_{DnaDamage}(t+1) = sgn[+\sigma_{Mutagen}(t) + \sigma_{ROS}(t)];$$

$$\sigma_{p53/Mdm2}(t+1) = sgn[+\sigma_{p53}(t) + \sigma_{Mdm2}(t) - 1];$$

$$\sigma_{AMP/ATP}(t+1) = sgn[-\sigma_{Nutrients}(t) + 1];$$

$$\sigma_{NF1}(t+1) = sgn[-\sigma_{PKC}(t) + 1];$$

$$\sigma_{PKC}(t+1) = sgn[+\sigma_{RTK}(t) + \sigma_{WNT}(t)];$$

$$\sigma_{RTK}(t+1) = sgn[+\sigma_{GFs}(t)];$$

$$\sigma_{RAGS}(t+1) = sgn[+\sigma_{Nutrients}(t) - \sigma_{Hypoxia}(t)];$$

$$\sigma_{Ras}(t+1) = sgn[-\sigma_{NF1}(t) + \sigma_{RTK}(t) + 1];$$

$$\sigma_{PI3K}(t+1) = sgn[+\sigma_{Ras}(t) + \sigma_{hTERT}(t)];$$

$$\sigma_{PTEN}(t+1) = 1;$$

$$\sigma_{PIP3}(t+1) = sgn[+\sigma_{PI3K}(t) - \sigma_{PTEN}(t) - \sigma_{p53/PTEN}(t) + 1];$$

$$\sigma_{PDK1}(t+1) = sgn[+\sigma_{PIP3}(t) + \sigma_{HIF1}(t) + \sigma_{Myc/Max}(t)];$$

$$\sigma_{IKK}(t+1) = sgn[+\sigma_{PKC}(t) + \sigma_{AKT}(t) + \sigma_{mTOR}(t) - \sigma_{PHDs}(t) - \sigma_{p53}(t) + \sigma_{TAK1}(t)];$$

$$\sigma_{NF-\kappa B}(t+1) = sgn[+\sigma_{PIP3}(t) + 2\sigma_{IKK}(t) - \sigma_{E-cadh}(t) + \sigma_{Snail}(t) - 1];$$

$$\sigma_{RAF}(t+1) = sgn[+\sigma_{PKC}(t) + \sigma_{Ras}(t)];$$

$$\sigma_{ERK}(t+1) = sgn[+\sigma_{RAF}(t)];$$

$$\sigma_{p90}(t+1) = sgn[+\sigma_{PDK1}(t) + \sigma_{ERK}(t)];$$

$$\sigma_{AKT}(t+1) = sgn[+\sigma_{PIP3}(t) + \sigma_{PDK1}(t) - 1];$$

$$\sigma_{WNT}(t+1) = sgn[-\sigma_{p53}(t) + \sigma_{Gli}(t)];$$

$$\sigma_{Dsh}(t+1) = sgn[+\sigma_{WNT}(t)];$$

$$\sigma_{APC}(t+1) = sgn[+\sigma_{PTEN}(t) + 1];$$

$$\sigma_{GSK-3}(t+1) = sgn[-\sigma_{p90}(t) - \sigma_{AKT}(t) - \sigma_{Dsh}(t) - \sigma_{mTOR}(t) + 3];$$

$$\sigma_{GSK-3/APC}(t+1) = sgn[+\sigma_{APC}(t) + \sigma_{GSK-3}(t) - 1];$$

$$\sigma_{\beta-cat}(t+1) = sgn[-\sigma_{GSK-3/APC}(t) - \sigma_{p53}(t) + 1];$$

$$\sigma_{Slug}(t+1) = sgn[-\sigma_{p53/Mdm2}(t) + \sigma_{NFkB}(t) + \sigma_{TCF}(t)];$$

$$\sigma_{mTOR}(t+1) = sgn[+\sigma_{RAGS}(t) + \sigma_{AKT}(t) + \sigma_{RHEB}(t) - \sigma_{AMPK}(t) - 1];$$

$$\begin{aligned}\sigma_{HIF1}(t+1) = \quad & sgn[+\sigma_{Hypoxia}(t) + \sigma_{mTOR}(t) - 2\sigma_{VHL}(t) - \sigma_{PHDs}(t) \\ & +\sigma_{Myc/Max}(t) - \sigma_{p53}(t) - \sigma_{FOXO}(t) + 2];\end{aligned}$$

$$\sigma_{COX412}(t+1) = sgn[+\sigma_{HIF1}(t)];$$

$$\sigma_{VHL}(t+1) = sgn[-\sigma_{Hypoxia}(t) - \sigma_{ROS}(t) + 1];$$

$$\sigma_{PHDs}(t+1) = sgn[-\sigma_{Hypoxia}(t) + \sigma_{ROS}(t) + 1];$$

$$\sigma_{Myc/Max}(t+1) = sgn[-\sigma_{TGF\beta}(t) + \sigma_{Myc}(t) + \sigma_{Max}(t) - \sigma_{MXI1}(t) - \sigma_{SmadE2F}(t) - 1];$$

$$\begin{aligned}\sigma_{Myc}(t+1) = \quad & sgn[+\sigma_{NF-\kappa B}(t) + \sigma_{ERK}(t) - \sigma_{HIF1}(t) + \sigma_{E2F}(t) \\ & +\sigma_{FosJun}(t) + \sigma_{TCF}(t) + \sigma_{Gli}(t) - 1];\end{aligned}$$

$$\sigma_{Max}(t+1) = 1;$$

$$\sigma_{MXI1}(t+1) = sgn[+\sigma_{HIF1}(t)];$$

$$\begin{aligned}\sigma_{TSC1/TSC2}(t+1) = \quad & sgn[-\sigma_{RAF}(t) - \sigma_{ERK}(t) - \sigma_{p90}(t) - \sigma_{AKT}(t) \\ & +\sigma_{HIF1}(t) + \sigma_{p53}(t) + \sigma_{AMPK}(t) + 1];\end{aligned}$$

$$\sigma_{RHEB}(t+1) = sgn[-\sigma_{TSC1/TSC2}(t) + 1];$$

$$\sigma_{p53}(t+1) = sgn[+\sigma_{HIF1}(t) - \sigma_{Bcl-2}(t) - \sigma_{Mdm2}(t) + \sigma_{CHK1/2}(t) + 1];$$

$$\sigma_{Bcl-2}(t+1) = sgn[+2\sigma_{NF-\kappa B}(t) - \sigma_{p53}(t) - \sigma_{BAX}(t) - \sigma_{BAD}(t)];$$

$$\sigma_{BAX}(t+1) = sgn[-\sigma_{HIF1}(t) + \sigma_{p53}(t) - \sigma_{Bcl-2}(t) + \sigma_{JNK}(t)];$$

$$\sigma_{BAD}(t+1) = sgn[-\sigma_{RAF}(t) - \sigma_{p90}(t) - \sigma_{AKT}(t) - \sigma_{HIF1}(t) + 1];$$

$$\sigma_{Bcl-X_L}(t+1) = sgn[-\sigma_{p53}(t) - \sigma_{BAD}(t) + 1];$$

$$\sigma_{Rb}(t+1) = sgn[-\sigma_{CycA}(t) - \sigma_{CycB}(t) - \sigma_{CycD}(t) - \sigma_{CycE}(t) - \sigma_{Mdm2}(t) + 2];$$

$$\sigma_{E2F}(t+1) = sgn[-2\sigma_{Rb}(t) - \sigma_{CycA}(t) - \sigma_{CycB}(t) + \sigma_{E2F}(t) + 1];$$

$$\sigma_{p14}(t+1) = sgn[+\sigma_{Ras}(t) + \sigma_{Myc/Max}(t) + \sigma_{E2F}(t) - 3];$$

$$\begin{aligned}\sigma_{CycA}(t+1) = \quad & sgn[+\sigma_{CycA}(t) - \sigma_{Rb}(t) - \sigma_{cdc20}(t) - \sigma_{p27}(t) \\ & -\sigma_{p21}(t) + \sigma_{E2F/CyclinE}(t) + \sigma_{cdh1/UbcH10}(t)];\end{aligned}$$

$$\sigma_{CycB}(t+1) = sgn[-\sigma_{p53}(t) - \sigma_{cdh1}(t) - \sigma_{cdc20}(t) - \sigma_{p27}(t) - \sigma_{p21}(t) + 1];$$

$$\begin{aligned}\sigma_{CycD}(t+1) = \quad & sgn[+\sigma_{NF-\kappa B}(t) - 2\sigma_{GSK-3}(t) + \sigma_{Myc/Max}(t) - \sigma_{p27}(t) - \sigma_{p21}(t) \\ & -\sigma_{p15}(t) - \sigma_{FOXO}(t) + \sigma_{FosJun}(t) + \sigma_{TCF}(t) + \sigma_{Gli}(t)];\end{aligned}$$

$$\sigma_{CycE}(t+1) = sgn[-\sigma_{Rb}(t) + \sigma_{E2F}(t) - \sigma_{CycA}(t) - \sigma_{p27}(t) - \sigma_{p21}(t)];$$

$$\sigma_{cdh1}(t+1) = sgn[-\sigma_{CycA}(t) - \sigma_{CycB}(t) + \sigma_{cdc20}(t) + 1];$$

$$\sigma_{cdc20}(t+1) = sgn[+\sigma_{CycB}(t) - \sigma_{cdh1}(t)];$$

$$\sigma_{UbcH10}(t+1) = sgn[+\sigma_{CycA}(t) + \sigma_{CycB}(t) - \sigma_{cdh1}(t) + \sigma_{cdc20}(t) + \sigma_{UbcH10}(t)];$$

$$\begin{aligned}\sigma_{p27}(t+1) = \quad & sgn[-\sigma_{AKT}(t) + \sigma_{HIF1}(t) - \sigma_{Myc/Max}(t) - \sigma_{CycA}(t) \\ & -\sigma_{CycB}(t) - \sigma_{CycD}(t) + \sigma_{SmadMiz-1}(t) + 1];\end{aligned}$$

$$\begin{aligned}\sigma_{p21}(t+1) = \quad & sgn[-\sigma_{AKT}(t) + \sigma_{HIF1}(t) - \sigma_{Myc/Max}(t) + \sigma_{p53}(t) \\ & +\sigma_{SmadMiz-1}(t) - \sigma_{hTERT}(t) + 1];\end{aligned}$$

$$\sigma_{Mdm2}(t+1) = sgn[+\sigma_{AKT}(t) + \sigma_{p53}(t) - \sigma_{p14}(t) - \sigma_{ATM/ATR}(t) + 1];$$

$$\sigma_{Smad}(t+1) = sgn[+\sigma_{TNF\alpha}(t) + \sigma_{TGF\beta}(t)];$$

$$\sigma_{SmadMiz-1}(t+1) = sgn[+\sigma_{Smad}(t) + \sigma_{Miz-1}(t) - 1];$$

$$\sigma_{SmadE2F}(t+1) = sgn[+\sigma_{Smad}(t)];$$

$$\sigma_{p15}(t+1) = sgn[+\sigma_{SmadMiz-1}(t) + \sigma_{Miz-1}(t)];$$

$$\sigma_{FADD}(t+1) = sgn[+\sigma_{TNF\alpha}(t)];$$

$$\sigma_{Caspase8}(t+1) = sgn[+\sigma_{FADD}(t)];$$

$$\sigma_{Bak}(t+1) = sgn[+\sigma_{Caspase8}(t)];$$

$$\sigma_{JNK}(t+1) = sgn[+\sigma_{TGF\beta}(t)];$$

$$\sigma_{FOXO}(t+1) = sgn[-\sigma_{AKT}(t) + 2];$$

$$\sigma_{FosJun}(t+1) = sgn[+\sigma_{ERK}(t) + \sigma_{JNK}(t)];$$

$$\sigma_{ROS}(t+1) = sgn[-\sigma_{COX412}(t) - \sigma_{GSH}(t)];$$

$$\sigma_{AMPK}(t+1) = sgn[-\sigma_{GFs}(t) + \sigma_{AMP/ATP}(t) + \sigma_{HIF1}(t) + \sigma_{ATM/ATR}(t) + 1];$$

$$\begin{aligned}\sigma_{Cytoc/APAF1}(t+1) = \quad & sgn[-\sigma_{AKT}(t) + \sigma_{p53}(t) - \sigma_{Bcl-2}(t) + \sigma_{BAX}(t) \\ & -\sigma_{Bcl-X_L}(t) + \sigma_{Caspase8}(t) + \sigma_{Bak}(t)];\end{aligned}$$

$$\sigma_{Caspase9}(t+1) = sgn[+\sigma_{Cytoc/APAF1}(t)];$$

$$\sigma_{Apoptosis}(t+1) = sgn[+\sigma_{Caspase8}(t) + \sigma_{Caspase9}(t)];$$

$$\sigma_{E-cadh}(t+1) = sgn[-\sigma_{NF-\kappa B}(t) - \sigma_{Slug}(t) - \sigma_{Snail}(t) + 3];$$

$$\sigma_{Glut-1}(t+1) = sgn[+\sigma_{AKT}(t) + \sigma_{HIF1}(t) + \sigma_{Myc/Max}(t) - 1];$$

$$\begin{aligned}\sigma_{hTERT}(t+1) = \quad & sgn[+\sigma_{NF1}(t) + \sigma_{NF-\kappa B}(t) + \sigma_{AKT}(t) + \sigma_{HIF1}(t) \\ & +\sigma_{Myc/Max}(t) - \sigma_{p53}(t) - \sigma_{SmadMiz-1}(t) - \sigma_{eEF2}(t) - 4];\end{aligned}$$

$$\sigma_{VEGF}(t+1) = sgn[+\sigma_{HIF1}(t) + \sigma_{Myc/Max}(t)];$$

$$\sigma_{E2F/CyclinE}(t+1) = sgn[+\sigma_{E2F}(t) + \sigma_{CycE}(t) - 1];$$

$$\sigma_{cdh1/UbcH10}(t+1) = sgn[+\sigma_{cdh1}(t) + \sigma_{UbcH10}(t) - 1];$$
$$\sigma_{TAK1}(t+1) = sgn[+\sigma_{TNFalpha}(t)];$$
$$\sigma_{GSH}(t+1) = sgn[+\sigma_{NF-\kappa B}(t) + \sigma_{Myc/Max}(t) + \sigma_{p21}(t)];$$
$$\sigma_{TCF}(t+1) = sgn[+\sigma_{\beta-cat}(t) - \sigma_{TAK1}(t)];$$
$$\sigma_{Miz-1}(t+1) = sgn[-\sigma_{Myc/Max}(t) + 1];$$
$$\sigma_{p70}(t+1) = sgn[+\sigma_{PDK1}(t) + \sigma_{mTOR}(t)];$$
$$\sigma_{ATM/ATR}(t+1) = sgn[+\sigma_{DnaDamage}(t)];$$
$$\sigma_{CHK1/2}(t+1) = sgn[+\sigma_{ATM/ATR}(t)];$$
$$\sigma_{DNARepair}(t+1) = sgn[+\sigma_{ATM/ATR}(t)];$$
$$\sigma_{eEF2K}(t+1) = sgn[+\sigma_{p90}(t) + \sigma_{p70}(t)];$$
$$\sigma_{eEF2}(t+1) = sgn[-\sigma_{eEF2K}(t) + 1];$$
$$\sigma_{p53/PTEN}(t+1) = sgn[+\sigma_{PTEN}(t) + \sigma_{p53}(t) - 1];$$
$$\sigma_{LDHA}(t+1) = sgn[+\sigma_{HIF1}(t) + \sigma_{Myc/Max}(t) - 1];$$
$$\sigma_{AcidLactic}(t+1) = sgn[+\sigma_{LDHA}(t)];$$
$$\sigma_{Snail}(t+1) = sgn[+\sigma_{NF-\kappa B}(t) - \sigma_{GSK-3}(t) - \sigma_{p53}(t) + \sigma_{Smad}(t) - 1];$$

Network attractors

In Tables S1 and S2 are listed all dynamical attractors exhibited by the network. They are grouped by microenvironmental conditions and the sizes of their basins of attraction, periods, and associated phenotype are provided. In Figure S1 the configurations comprising all the fixed points and limit cycles associated to five distinct microenvironments are shown.

The similarity between network attractors were analyzed through their average Hamming distance d_H. For two distinct fixed points, d_H is simply the fraction of corresponding nodes exhibiting distinct activities, whereas for two limit cycles d_H was determined as the average value of all pairwise Hamming distance between their states. Fixed the group (microenvironment), their proliferative attractors are very similar with $d_H = 0.084$ in average. The same is true for quiescent attractors with an average intragroup $d_H = 0.097$. For apoptotic attractors, the intragroup distance is $d_H = 0.270$ in average, indicating protein activation patterns significantly distinct. Finally, the Hamming distances between apoptotic, proliferative and quiescent attractors are higher ($0.275 \leq d_H \leq 0.49$), both inter and intragroup, indicating clearly distinct cell responses.

Driver mutations

Considering that DNA integrity sensors are defective, leading to the permanent inactivation of the node DNA damage, the additional driver mutations that confer either proliferative advantage or the capacity to evade apoptosis are listed in tables S3 and S4.
